# Supplementary material for: Determining the defining lengths between mature microRNAs/small interfering RNAs and tinyRNAs
Source: Sci Rep. 2023 Nov 13;13:19761. doi: 10.1038/s41598-023-46562-6 (PMC10643408; doi:10.1038/s41598-023-46562-6)
Supplement: Supplementary file 1 — Supplementary Figures. [file 41598_2023_46562_MOESM1_ESM.docx]

**SUPPLEMENTAL DATA**

**Determining the defining lengths between mature microRNAs/small interfering RNAs and tinyRNAs**

GeunYoung Sim, Audrey C. Kehling, Mi Seul Park, Cameron Divoky, Huaqun Zhang, Nipun Malhotra, Jackson Secor and Kotaro Nakanishi

**
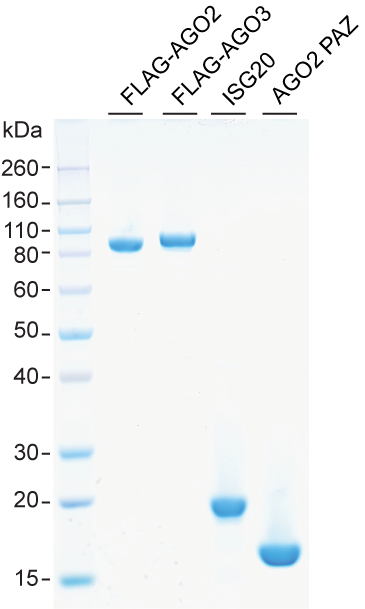
**

**Supplementary Figure S1.** SDS-PAGE analysis of the purified recombinant proteins used in this study. The protein bands were visualized by Coomassie brilliant blue staining.

**
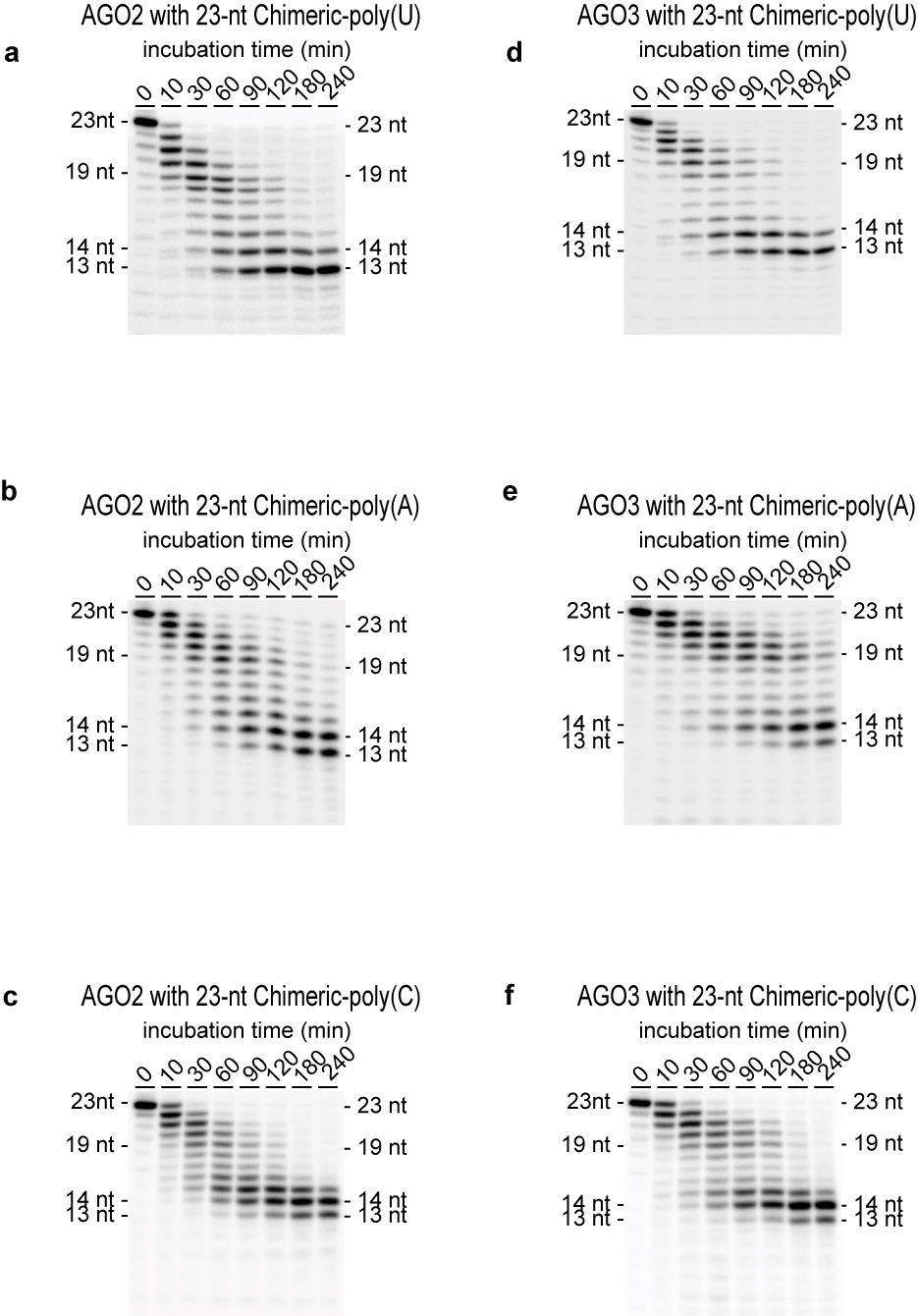
**

**Supplementary Figure S2.** Representative gel images of *in vitro* trimming assays. (**a-c**) FLAG-AGO2 was programmed with either chimeric poly(U) (in panel **a**), chimeric poly(A) (in panel **b**), or chimeric poly(C) (in panel **c**). (**d-f**) FLAG-AGO3 was programmed with either chimeric poly(U) (in panel **d**), chimeric poly(A) (in panel **e**), or chimeric poly(C) (in panel **f**).

**
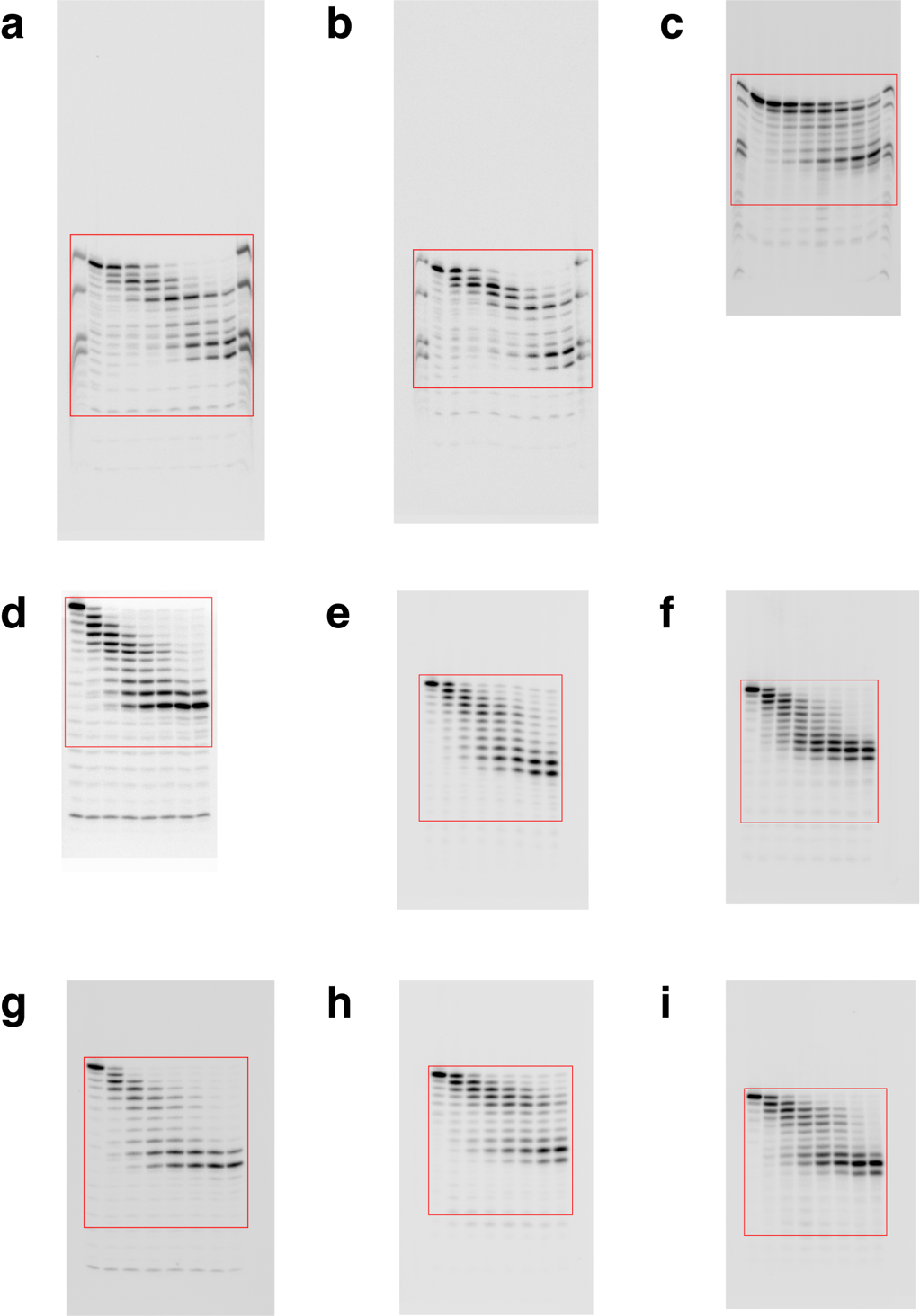
**

**Supplementary Figure S3.** Full denaturing gel images. (**a-b**) Denaturing gel images for time-course *in vitro* trimming assay of a 5’-end radiolabeled 23-nt miR-20a loaded into FLAG-AGO2 for Fig.2b (in panel **a**), and -AGO3 for Fig. 2c (in panel **b**). A denaturing gel image for time-course *in vitro* trimming assay of a 5’-end radiolabeled 21-nt let-7a loaded into FLAG-AGO3 for Fig.2d (in panel **c**). (**d-f**) Denaturing gel images for time-course *in vitro* trimming assay of FLAG-AGO2 programmed with a 5’-end radiolabeled 23-nt Chimeric-poly(U) for Fig. S2a (in panel **d**), with a 5’-end radiolabeled 23-nt Chimeric-poly(A) for Fig. S2b (in panel **e**), and with a 5’-end radiolabeled 23-nt Chimeric-poly(C) for Fig. S2c (in panel **f**). (**g-i**) Denaturing gel images for time-course *in vitro* trimming assay of FLAG-AGO3 programmed with a 5’-end radiolabeled 23-nt Chimeric-poly(U) for Fig. S2d (in panel **g**), with a 5’-end radiolabeled 23-nt Chimeric-poly(A) for Fig. S2e (in panel **h**), and with a 5’-end radiolabeled 23-nt Chimeric-poly(C) for Fig. S2f (in panel **i**). The regions of the original gels used in the main figures and supplementary figures are indicated by red boxes.


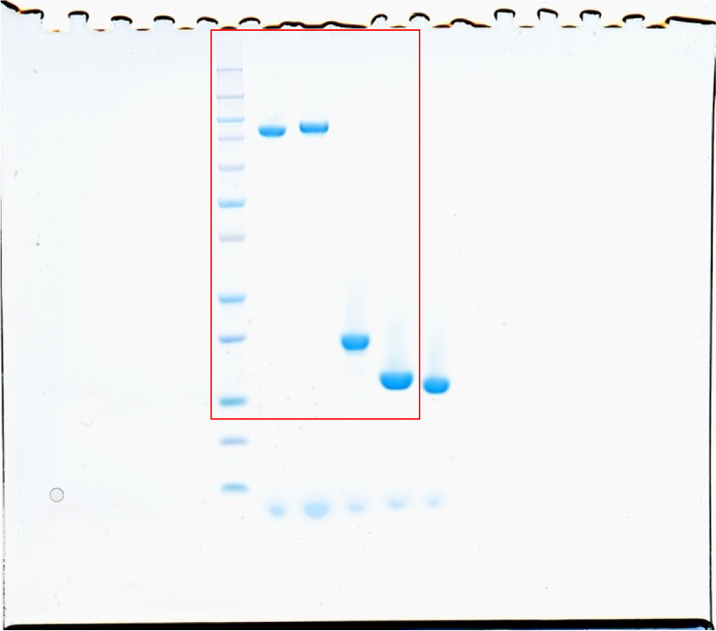


**Supplementary Figure S4.** Full SDS-PAGE analysis. A SDS-PAGE gel image of FLAG-AGO2, FLAG-AGO3, ISG20, and AGO2 PAZ for Fig. S1. The region of the original gel used in the Fig. S1 is indicated by a red box.
